# Whisking and locomotion are jointly represented in superior colliculus neurons

**DOI:** 10.1371/journal.pbio.3003087

**Published:** 2025-04-07

**Authors:** Suma Chinta, Scott R. Pluta

**Affiliations:** Department of Biological Sciences, Purdue University, West Lafayette, Indiana, United States of America; International School for Advanced Studies, ITALY

## Abstract

Active sensation requires the brain to interpret external stimuli against an ongoing estimate of body position. While internal estimates of body position are often ascribed to the cerebral cortex, we examined the midbrain superior colliculus (SC), due to its close relationship with the sensory periphery as well as higher, motor-related brain regions. Using high-density electrophysiology and movement tracking, we discovered that the on-going kinematics of whisker motion and locomotion speed accurately predict the firing rate of mouse SC neurons. Neural activity was best predicted by movements occurring either in the past, present, or future, indicating that the SC population continuously estimates a trajectory of self-motion. A combined representation of slow and fast whisking features predicted absolute whisker angle at high temporal resolution. Sensory reafference played at least a partial role in shaping this feature tuning. Taken together, these data indicate that the SC contains a joint representation of whisking and locomotor features that is potentially useful in guiding complex orienting movements involving the face and limbs.

## Abstract 2

To navigate the world, the brain needs to have internal models of self-motion, for example of whisker movement in mice. This study shows how neurons in the midbrain superior colliculus encode internal and external signals to build an accurate representation of whisker position and locomotion speed.

## Introduction

Internal models of self-motion play a crucial role in our ability to navigate the world [1]. They help us estimate the relative distance of stimuli by constantly monitoring the position and movement of our sensors. In a scenario absent of body movement, object localization is performed by mapping stimuli directly to receptors on the body surface. The somatosensory whisker system of rodents contains an elegant map of receptors, where each whisker on the face activates a corresponding coordinate in the brain [2–8]. However, in real life, the position of the whiskers constantly changes as animals navigate their environment and orient toward stimuli [9,10]. Therefore, active sensing requires the brain to maintain an ongoing representation of self-motion [11].

The midbrain superior colliculus (SC) contains multiple maps of sensory space and is hypothesized to play an essential role in controlling whisking and locomotor movements [12–15]. It receives monosynaptic input from the vibrissae regions of the trigeminal nucleus, somatosensory, and motor cortices, as well as the cerebellum, making it an ideal candidate for sensing and generating movement signals [16–20]. Movements are partially encoded in the whisker system through reafference: the self-generated activation of receptors in the follicle [21]. During rhythmic whisking, neurons in the trigeminal ganglion, brainstem, thalamus, and cortex encode the relative position of the whisker within a movement cycle, termed phase [22–27]. SC neurons are known to briefly spike at the onset of whisking induced by facial nerve stimulation, but their functional relationship to phase is unknown [28]. Since phase information represents the *relative* position of the whisker, whisk offset and amplitude information are necessary to build a complete model of absolute whisker angle [11,13,29]. SC neurons could conceivably use or build such model, since they receive input from phase, amplitude, and offset encoding brain areas [19,23,29–36]. Despite these known anatomical connections, the relationship between SC spiking and volitional whisker position is unknown.

The SC is thought to directly control whisker movement, since a sparse population of its excitatory neurons target the downstream nucleus that drives the whisking muscles [17,37,38]. Functional evidence supporting a role for the SC in whisker movement comes from its electrical activation under anesthesia, which causes sustained whisker protraction [39,40]. Likewise, unilateral ablation of the SC retracts the resting position of the contralateral whisker pad but has little effect on volitional bouts of whisking [37]. These data argue that in the absence of whisking, SC spiking is directly related to whisker protraction. However, recent evidence reveals that SC neurons also have excitatory connections with the nucleus that mediates whisker retraction [37,38,41]. Ultimately, the relationship between SC activity and volitional whisker position remains unclear. Given its diversity of afferent and efferent inputs, we hypothesized that SC neurons accurately predict whisker position at high temporal resolution.

To determine the kinematic features encoded by SC neurons, we performed high-density electrophysiology and high-speed videography in head-fixed, freely whisking, and locomoting mice. We discovered that the firing rates of many SC neurons were predicted by a single or multiple kinematic features. SC activity was predicted by movements occurring either in the past, present, or future time domains. Nearly half of all SC neurons that were tuned to whisking offset and/or amplitude were also tuned to whisking phase. By combining these features, SC neurons computed absolute whisker angle at high temporal resolution.

## Results

### The firing rates of SC neurons are linearly related to self-motion

**Fig 1:**
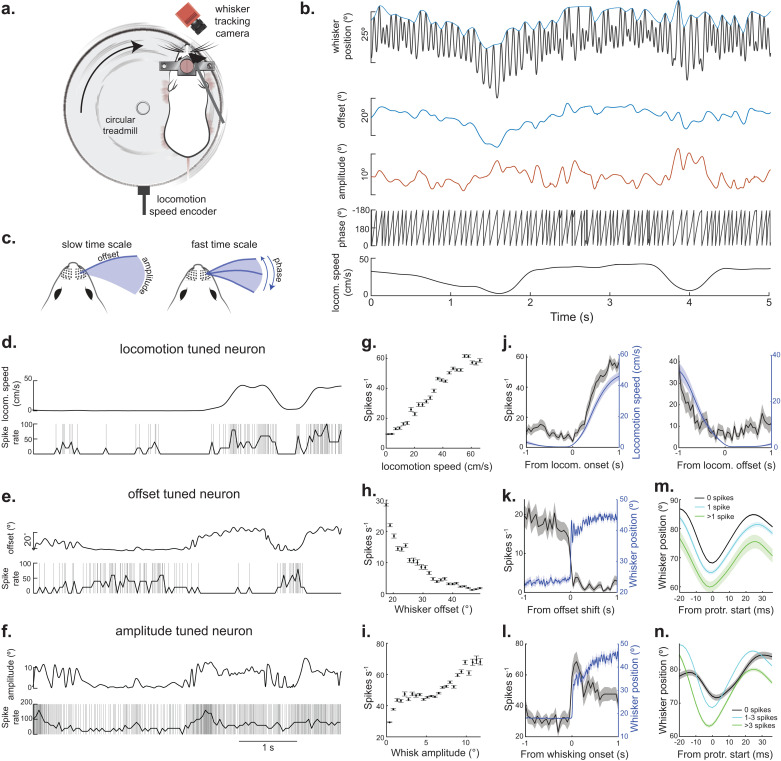
The firing rate of SC neurons is linearly related to self-motion. **(A)** A schematic of the experimental setup illustrating a head-fixed mouse locomoting on a circular treadmill and actively whisking in air. A high-speed camera and digital encoder recorded whisker position and locomotion speed, respectively. **(B)** Example traces of whisking kinematics and locomotion speed over time. Whisking features midpoint, amplitude, and phase were calculated from angle. **(C)** Illustration displaying the slow-changing features whisker midpoint and amplitude (left) and the fast-changing feature whisker phase (right). **(D–F)** The relationship between self-motion (locomotion (D), midpoint (E), or amplitude (F)) and spike rate in three example neurons. Each row of plots corresponds to a different neuron. **(G)** Firing rate as a function of locomotion speed for the neuron in panel D. **(H)** Firing rate as a function of whisker midpoint for the neuron in panel E. **(I)** Firing rate as a function of whisk amplitude for the neuron in panel F. **(J)** Neuronal firing rate aligned to locomotion onset and offset (left and right) for the neuron in panel D. **(K)** Whisker position (blue) and firing rate (black) aligned to when the mouse made a large increase in midpoint. **(L)** Firing rate (black) aligned to the onset of whisking (blue) for the neuron in panel F. **(M)** Mean whisker position (aligned to protraction start) preceded by either 0, 1, or >1 spike in the neuron in panel E. **(N)** Mean whisker position (aligned to protraction start) preceded by either 0, 1–3, or >3 spikes in the neuron in panel F.

To reveal the neural representation of whisking and locomotion, we recorded single-unit activity from populations of neurons located in the deep layers of the lateral SC (Fig 1A and S1A and S1G). Mice were head-fixed yet able to whisk and locomote freely on a treadmill, allowing for behaviors that were under complete volitional control [42–45]. The right eye was covered with an opaque object, to block potential visual stimulation. In an average recording session, we imaged whisking for 600 ± 167 s, where mice completed 5,857 ± 1,507 whisk cycles and locomoted 103 ± 28 m across a wide range of speeds (S1B Fig). Using high-speed (500 fps) infrared imaging and markerless tracking [46], we calculated whisker position over time and decomposed its movement into distinct kinematic features, known as offset, amplitude, and phase (Fig 1B, see Methods). We categorized these whisking features as ‘slow’ or ‘fast’, based on the derivative of their autocorrelations (S1C–S1F Fig), and deemed whisker offset and amplitude as slow, relative to the faster (<1 whisk cycle) movements that underlie phase (Fig 1C).

To determine the relationship between SC spiking and the slow kinematic features (including locomotion), we first used a traditional tuning curve approach. In many neurons, we observed a linear relationship between firing rate and the kinematics of whisking and locomotion speed (Fig 1D–1I and S1 Video). To better understand the temporal fidelity of this relationship, we plotted firing rate relative to transitions in self-motion (locomotion onset/offset, shifts in whisk offset, whisking onset), revealing precise and accurate coupling between spike timing and behavior (Fig 1J–1L). Furthermore, we segregated whisks according to the number of spikes in the preceding full whisk cycle (Fig 1M and 1N) and found that the number of preceding spikes was correlated to the offset/amplitude in the upcoming whisk. Taken together, these data highlight the strong and temporally precise correlation between the activity of SC neurons and self-motion. While this analysis provides an intuitive basis for understanding the relationship between neural activity and behavior, it fails to capture nonlinear dynamics or address the inherent correlation between kinematic features. To address these issues and disentangle the dependence of SC spiking on the different features of self-motion, we implemented a previously established linear-nonlinear Poisson (LNP) spiking GLM [24,47–49]. The LNP model in combination with forward search is advantageous because it identifies the kinematic feature(s) that provide(s) a significant contribution to spike prediction (S2 Fig).

### Mixed neuronal selectivity for whisking and locomotion

**Fig 2:**
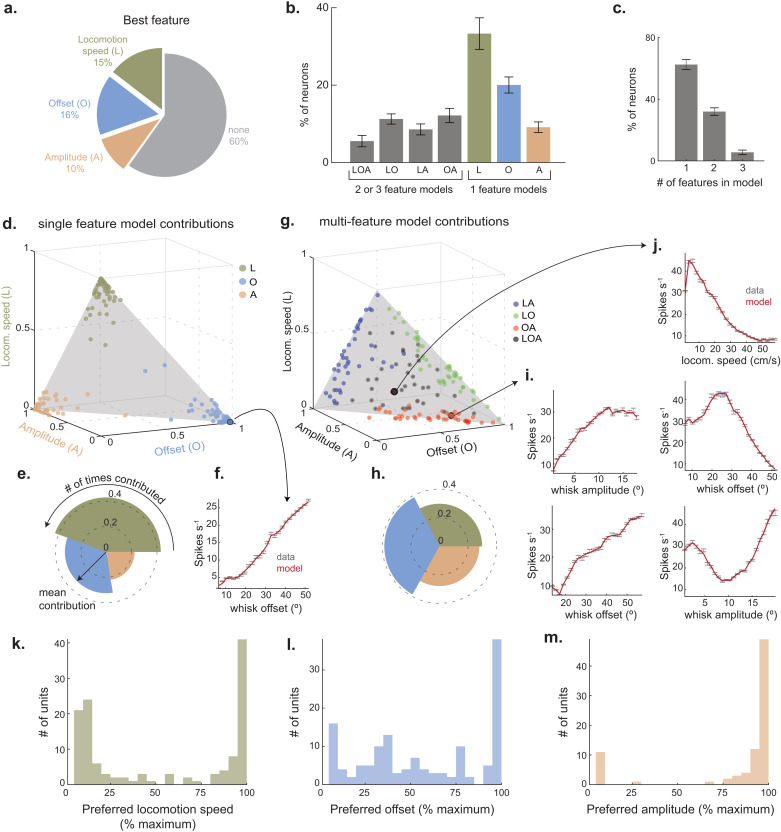
Single neuron encoding of whisking and locomotion. **(A)** The kinematic feature that best predicted spiking across the recorded population (feature with largest log-likelihood among single feature models). **(B)** Percentage of neurons with single and multi-feature selectivity for whisking and locomotion (352/932 neurons, 12 mice). **(C)** Percentage of neurons selective to 1, 2, or 3 kinematic features (352 neurons). **(D)** Scatterplot of normalized contributions of individual features (locomotion speed, midpoint, and amplitude) to selected model of single-feature selective neurons (187 neurons). **(E)** Polar plot denoting the relative contribution of individual features to the full model for single-feature selective neurons (187 neurons). **(F)** Neuronal tuning curve to midpoint (best feature selected by model). **(G)** Scatterplot of normalized contributions of individual features to selected model of multi-feature selective neurons (165 neurons). **(H)** Polar plot denoting the relative contribution of individual features to the full model for multi-feature neurons (165 neurons). **(I, J)** Tuning curves of two neurons with significant contributions from multiple kinematic features. **(K–M)** Histogram of the preferred locomotion speed, whisk offset, and amplitude, for neurons that were best predicted by that feature (125 neurons whose best feature was locomotion speed; 136 neurons whose best feature was midpoint; 83 neurons whose best feature was amplitude). The individual values for panels B and C are included in S1 Data, Fig 2 sheet.

To identify the self-motion features that predict SC spiking, we fit every neuron with single- and multi-feature LNP models. Using the forward search approach, we identified the model with the least number of features that best predicted spiking. Any kinematic feature that did not significantly boost model performance over the best single-feature model was discarded (S2C–S2E Fig). This approach revealed that 40% of SC neurons had a firing rate predicted by one or more slow self-motion features (345/859 neurons in 12 mice, Fig 2A and 2B). The activity of most neurons was best predicted by whisk offset or locomotion speed (16% and 15% of neurons, respectively), while a smaller subset of neurons preferred whisk amplitude (10%). For most neurons tuned to self-motion (59%, 180/345), only a single kinematic feature provided a significant contribution to spike prediction, while prediction accuracy in the remaining neurons benefited from a combination of features (41%, 165/345, Fig 2B and 2C). Among the single-feature neurons, locomotion speed was most often the best predictor of spiking (Fig 2D–2F). However, among neurons with multi-feature tuning, whisk offset emerged as the strongest (Fig 2G and 2H). These multi-feature neurons could help build an accurate model of whisker position (as in offset + amplitude neurons: MA) or enable coordinated adjustments to whisking and locomotion (as in locomotion + X neurons: LM, LA, or LMA). Across the population, SC neurons preferred a wide range locomotion speeds and whisk offsets (Fig 2K and 2L), suggesting that SC neurons potentially play a complex role in these movements. Conversely, SC neurons most often preferred the largest whisk amplitudes (Fig 2M), suggesting that SC spiking could facilitate large, exploratory movements [50].

### Past, present, and future movements predict SC spiking

**Fig 3:**
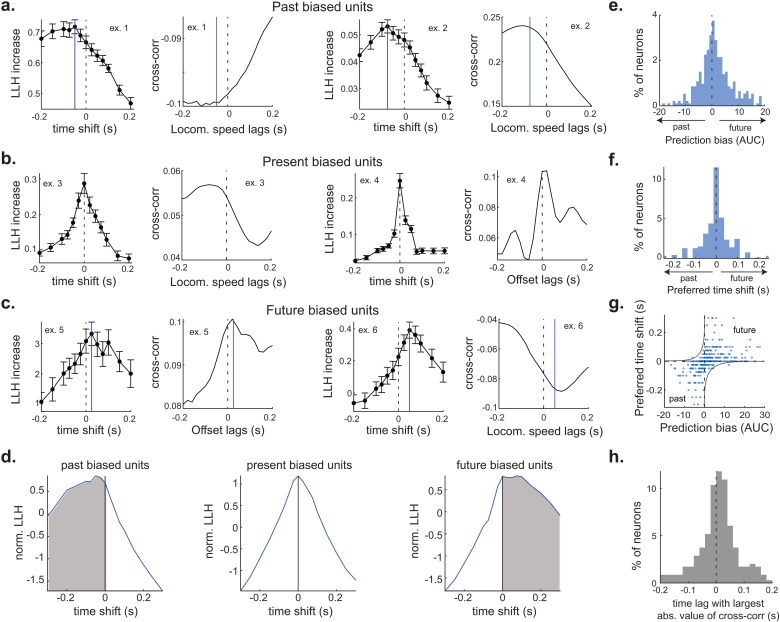
The SC encodes past, present, and future movements. **(A)** Two example neurons with firing rates more accurately predicted by running speeds in the past. Left panel, the *y*-axis measures the log-likelihood of spike prediction, which reflects the accuracy of the neural encoding model. The *x*-axis displays the time shift of the self-motion feature relative to the onset of neural activity, ranging from −0.2 s (indicative of the past) to +0.2 s (indicative of the future) Right panel, the cross-correlation between the preferred movement feature and neuronal firing rate. **(B, C)** Four more example neurons with spiking most accurately predicted by present (B) or future (C) self-motion features. All error bars represent mean ± standard error. **(D)** Unsupervised clustering of time-shifted neuronal encoding curves created three clusters that segregate into past, present, and future biased units (78, 123, and 104 neurons, respectively). The templates of these clusters are displayed. **(E)** Distribution of temporal biases which measures the area under the curve for future times relative to past times (12 mice, 347 neurons). **(F)** Distribution of preferred time shifts (12 mice, 347 neurons). **(G)** Relationship between temporal bias and preferred time shift (12 mice, 347 neurons). **(H)** The time lag with the largest absolute cross-correlation between each neuron’s preferred kinematic feature and its firing rate. The kinematic preference of each neuron was determined by the linear-nonlinear Poisson model.

Active sensing is a recurrent process that integrates knowledge of the body’s current state with future predictions [9,51,52]. Such computations are critical for adapting movement trajectories based on environmental cues [10,12,42,53]. To determine if the SC is capable of this computation, we utilized time-shifted models and tested the preference of SC neurons for past, present, and future kinematic features. Each neuron was fitted with time-shifted models using the single kinematic feature that was its best predictor. To complement this analysis, we also cross-correlated SC firing rates with their preferred kinematic feature. SC neurons displayed one of three temporal preferences. Units biased toward the past exhibited relatively steady model performance for past time shifts, contrasted with a rapid decay in performance for the future (Fig 3A). In some neurons, peak model performance also occurred in the past, underscoring their prominent role in storing traces of prior motion. Present biased units showed a symmetric decline in model performance, reflecting a real-time representation of self-motion (Fig 3B). In these units, spike timing suggests a role either in immediate sensory feedback or an efferent copy of motor commands. Future biased units displayed steady model performance for future time shifts but a rapid decay for the past (Fig 3C). This pattern implies a predictive model of future body position. Unsupervised (k-means) clustering of neuron response profiles confirmed these categories, distinguishing three principal clusters that broadly corresponded to past, present, and future biased populations (Fig 3D). The distribution of temporal biases and preferred time shifts indicate that the SC population represents a trajectory of body movement that spans hundreds of milliseconds (Fig 3E–3G). The temporal bias of neurons only partially varied according to their kinematic feature preference, with offset encoding neurons having a small, but significant, bias for future time points (S3B Fig). A large fraction of SC neurons preferred time-shifts within 50 ms of the present, a timescale relevant to whisking (mean whisk period is 52 ms). The largest cross-correlation (absolute value) between the preferred kinematic feature and firing rate revealed a similar distribution of temporal preferences (Fig 3H).

Next, we analyzed the temporal and feature preference of neurons using a single model that encompassed past, present, and future movements, to reduce temporal prediction biases caused by autocorrelations (S4 Fig). Using this approach on each kinematic feature separately, we found that whisk offset was most often the best predictor of SC spiking, while a minority of neurons were best predicted by whisk amplitude or locomotion speed (S4 Fig). The preferred time window was often within 50 ms of the present, but periods further in the past and future were also found. Overall, these data further support the notion that the SC, similar to other brain areas, represents of a trajectory of movements that span hundreds of milliseconds [54–56].

### The SC contains a comprehensive map of whisker phase

**Fig 4:**
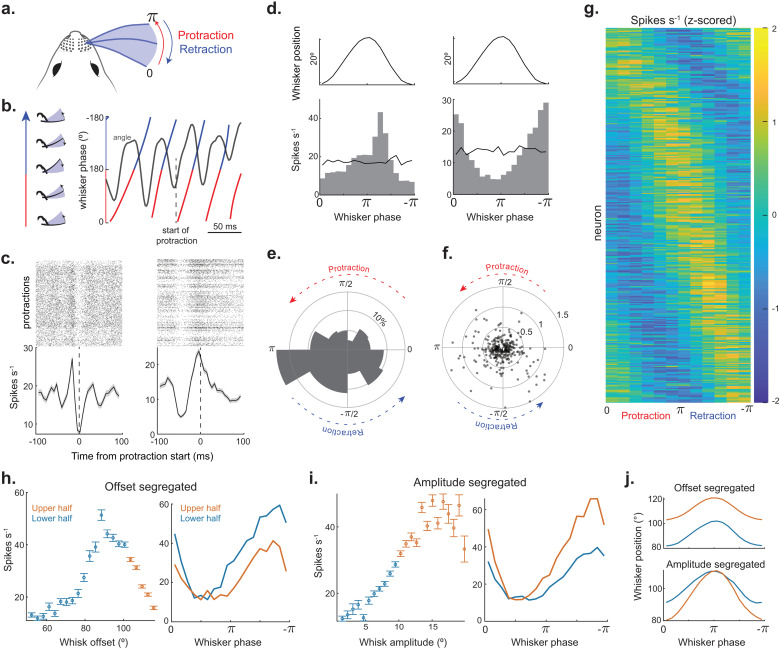
SC neurons encode the phase of whisker motion in combination with slow self-motion features. **(A)** Schematic of relationship between whisker position and phase. Protraction period of the whisk cycle is represented in red and the retraction period in blue. **(B)** Aligned traces of whisker position (in gray) and whisker phase (color). Dashed line represents the start of protraction in a whisk cycle. **(C)** Spike raster (top) and mean firing rate (bottom) aligned to the start of whisker protraction for two example neurons. **(D)** Top: Whisker position as a function of phase. Bottom: Phase tuning curves for the two neurons in panel C. The solid black line indicates the neuron’s firing rate if spike times were randomly distributed. **(E)** Polar histogram of preferred phase angle across the population of significantly tuned neurons (*p* < 0.05, 12 mice, 363 neurons, Rayleigh test). **(F)** Preferred phase and modulation depth for each neuron (363 neurons). **(G)** Heat map of phase tuning curves aligned by their preferred phase (12 mice, 363 neurons). **(H)** Left: Whisk offset tuning for an example neuron that encodes whisk offset, amplitude, and phase. Offsets were segregated into upper (orange) and lower (blue) halves. Right: Whisker phase tuning of the neuron in panel H segregated by upper and lower offsets. **(I)** Left: Whisker amplitude tuning for neuron in panel H color-coded for upper and lower halves. Right: Whisker phase tuning of the neuron in panel H segregated by upper and lower whisk amplitudes. **(J)** Whisker position as a function of phase for offset (top) and amplitude segregated (bottom) whisk cycles for the example neurons in panels H and I.

In exploring the representation of self-motion, we extended our analysis beyond the slow-changing features to investigate neural selectivity to the phase of whisker motion. Phase tuning has been observed throughout the ascending pathway of the somatosensory whisker system, but it has never before been investigated in the SC [22,24–26]. To examine phase tuning in SC neurons, we aligned spikes to the onset of whisker protraction, and we also generated spike triggered averages of phase angle using an established approach (Fig 4A–4D) [23,24]. Many neurons displayed non-uniform firing rates, often having a clear preference for either the protraction or retraction period of the whisk cycle. Across the population, 39% of neurons were significantly tuned to phase (363/932, Rayleigh test, *p* < 0.05), with the most frequent phase preferences occurring during the retraction period (Fig 4E and 4F). Nonetheless, the overall distribution of neuronal preferences covered the entire range of phase angles (Fig 4G). Half of all neurons tuned to phase also encoded one or more slow self-motion features (53%, 191/363). This is exemplified in Fig 4H and 4I, by showcasing a neuron with phase tuning modulated by whisk offset and amplitude. We demonstrate this slow-feature scaling of the phase response by evenly dividing phase angles between their upper and lower percentiles of offset or amplitude (Fig 4J). Among the 191 phase tuned units that also encoded one or more slow self-motion features, 113 units encoded locomotion speed, 106 units encoded whisker offset, and 93 units encoded whisker amplitude. Overall, 93 phase-sensitive units encoded a single slow feature, while 98 units encoded multiple slow features. This fast + slow coding scheme theoretically supports the computation of absolute whisker angle. We experimentally tested this theory below.

### The SC computes the absolute angle of whisker position

**Fig 5:**
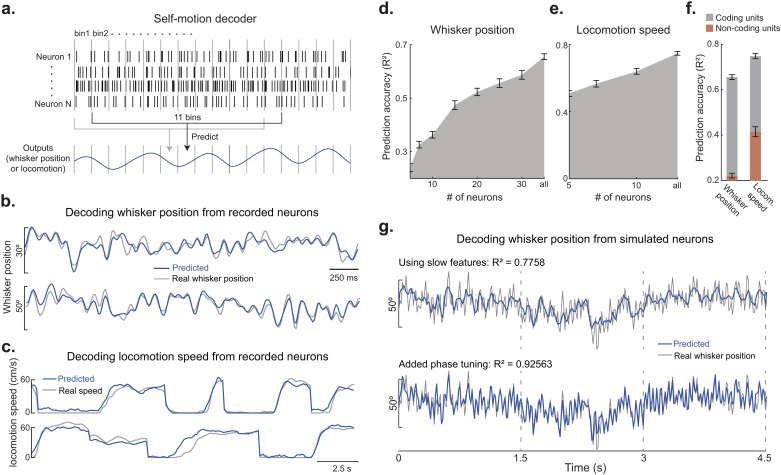
SC spiking accurately predicts whisker angle and locomotion speed. **(A)** Schematic of self-motion decoder. To predict the self-motion feature in each time bin (15 ms for whisker position and 100 ms for locomotion speed), the firing rate of N neurons in *T* time bins was used (*T* = 11 for whisker position, 9 for locomotion speed). **(B)** Prediction of whisker position from all whisking selective neurons (as determined by linear-nonlinear Poisson model) in a recording session (49 neurons, *R*^2^ = 0.72 mouse 1; 35 neurons, *R*^2^ = 0.66, mouse 2). **(C)** Prediction of locomotion speed from all locomotion selective neurons in a recording session (16 neurons, *R*^2^ = 0.72, mouse 1; 11 neurons, *R*^2^ = 0.82, mouse 2). **(D**, **E)** Prediction accuracy of whisker position (in D) and locomotion speed (in E) as a function of increasing number of neurons in the decoder model (7 mice in D, and 10 mice in E). Only neurons with significant self-motion tuning were used. **(F)** Prediction accuracy of whisker position and locomotion speed for self-motion coding and non-coding units (12 mice). **(G)** Top, decoding whisker position from simulated neurons that only encode slow self-motion features (*n* = 40 neurons). Bottom, decoding whisker position after the addition of simulated neurons that are tuned to whisker phase (bottom) (*n* = 40 neurons, 20 encode slow self-motion, 20 encode phase tuning). The individual values for panels D, E, and F are included in S1 Data, Fig 5 sheet.

We implemented a decoder model based on ridge regression to predict absolute whisker angle from SC population activity spanning past, present, and future time bins (Fig 5A, see Methods). For each 175-ms period of SC activity, whisker position was predicted in a 15-ms window, to allow for the capture of rapid, sub-cycle changes in whisker position. Remarkably, whisker position was predicted with a high level of accuracy, as evidenced in snapshots from two example mice (Fig 5B) and overall model performance across the population of mice (Fig 5D and S5). To our knowledge, the accuracy of this result surpasses other tested brain regions. However, given the uniquely large size of our dataset, encompassing several thousand whisk cycles in each mouse, such comparisons are difficult to interpret. Interestingly, SC activity was also adept at decoding locomotion speed (Fig 5C). Decoder performance was robust, even with a modestly sized population of units, and performance gradually increased with the number of neurons included in the model (Fig 5D and 5E). When we used a sub-sample of the population to estimate prediction accuracy, we randomly selected self-motion encoding neurons, and the randomization was repeated 10 times to get a more representative estimate. Neurons in our recorded population that did not encode self-motion (as determined by the LNP model) provided very little predictive power for whisker position (Fig 5F, orange bars). To disentangle the impact of slow and fast (phase) kinematic tuning on decoding whisker position, we simulated the activity of multiple neurons that closely mirrored our experimental observations (see Methods). Results from this simulation revealed that neurons tuned exclusively to slow self-motion features (offset and amplitude) can reliably predict gradual changes in whisker position. Next, by adding neurons with phase tuning, model performance was markedly enhanced, by capturing the rapid, sub-cycle changes in whisker position (Fig 5G, *R*^2^ = 0.92 versus *R*^2^ = −0.1 for shuffled spikes in S5E Fig). To identify the theoretical upper limit of model performance, we arithmetically calculated whisker angle from our decomposed offsets, amplitudes, and phases, and found that the arithmetic calculation was almost perfectly correlated to our experimental measure of angle (*R*^2^ = 0.95). Taken together, these data illustrate the importance of phase tuning and the complementary nature of slow and fast self-motion tuning for generating a high-resolution map of absolute whisker angle in the SC.

### The representation of self-motion is modified by sensory reafference

**Fig 6:**
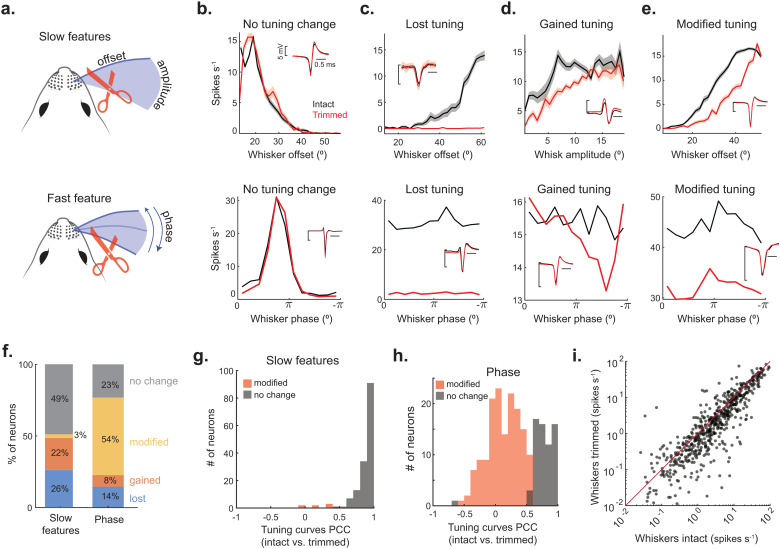
The representation of self-motion is modified by sensory reafference. **(A)** A schematic showing whisker trimming and self-motion features. **(B–E)** Top: Four example tuning curves for slow self-motion features during free whisking (black) and after trimming off the whiskers (red). Insets are spike waveforms during the two conditions. Bottom: Four example tuning curves to whisker phase during free whisking (black) and after trimming (red). **(F)** Percent of units that lost, gained, modified, or had no change in slow- or fast-feature tuning after whisker trimming (slow: 8 mice, 301 neurons; phase: 8 mice, 280 neurons). **(G)** Pearson correlation coefficient (PCC) between the slow feature tuning curves constructed before and after trimming. The tuning curves representing the best movement feature were correlated. Units in red have significantly modified phase tuning curves (PCC, *p* < 0.05, 8 neurons; no change, *p* > 0.05, 147 neurons). (H) PCC between phase tuning curves calculated before and after whisker trimming. Units in red have significantly modified phase tuning curves (PCC, *p* < 0.05, 152 neurons; no change, *p* > 0.05, 65 neurons). **(I)** Firing rates of SC neurons during whisking and locomotion calculated before and after whisker trimming. The whisker position and locomotion speed occupancies were selected to be equivalent between the conditions (8 mice, 636 neurons, Wilcoxon signed rank test, *p* = 2e^−26^).

To determine if sensory reafference plays a significant role in the representation of self-motion, we trimmed the length of the whiskers (to less ≤4 mm), significantly diminishing the inertial forces on the follicle [22] (Fig 6A and S2 and S3 Videos). To determine the impact of trimming on the different slow kinematic features, we calculated LNP model performance pre- and post-trimming. To control for changes in behavior between the trimming conditions, we only compared model performance across equivalent ranges of whisking and locomotion. A notable portion of neurons with significant model performance pre-trimming maintained significant model performance post-trimming. To determine if these neurons displayed a significant change in tuning shape, we calculated the Pearson correlation between their pre- and post-trimming tuning curves. Only a small fraction (3%) of these neurons exhibited a significant modification to tuning shape (Fig 6B and 6E–6G). Nearly half (49%) of all neurons maintained significant model performance post-trimming and did not change their tuning shape. In other neurons, LNP model performance decreased to chance post-trimming, indicating that these neurons lost their ability to encode self-motion (26%, Fig 6C and 6F). Other neurons gained significant model performance post-trimming (22%, Fig 6D and 6F). To test the influence of reafference on phase tuning, we calculated the Pearson correlation between the pre- and post-trimming tuning curves of each neuron (Fig 6H). Overall, whisker trimming had a more consistent effect on phase tuning, with 54% of all phase-tuned neurons exhibiting a significant modification in tuning shape (Fig 6C–6E, bottom row and F). Only 23% of all phase-tuned neurons displayed no change in tuning after trimming (Fig 6F and 6G). Overall, trimming caused a significant reduction in firing rates, even when controlling for whisker position and locomotion speed occupancies between the conditions (Fig 6I, 8 mice, 636 neurons, Wilcoxon signed rank test, *p* = 2e^−26^). Even though a range of 1–6 whiskers were intact pre-trimming (mean of 3 intact whiskers), the number of neurons with a tuning change post-trimming was uncorrelated to this initial quantity. Overall, these data reveal that sensory reafference at least partially shapes the representation of self-motion in SC neurons.

## Discussion

We discovered that the kinematics of active whisking and locomotion speed accurately predict spiking in SC neurons. Our analysis revealed populations of neurons that were maximally responsive to movements either in the past, present, or future. Therefore, the SC creates a continuous sensorimotor representation of movement trajectories. This internal monitoring of whisking operated across timescales, with half of all self-motion neurons encoding a combination of slow and fast kinematic features. This comprehensive map of self-motion accurately predicted absolute whisker angle. Sensory reafference played at least a partial role in building this model. Many studies have demonstrated that locomotion modulates neural activity, but how this modulation changes as a function of speed has often been overlooked [57–65]. Our dataset, collected across a wide range of locomotor speeds, revealed a diversity of tuning profiles. SC firing rates have been known to increase at the onset of locomotion [66], but we found a broad range of preferences (slow and fast speeds) that predicted locomotion speed with incredible accuracy. Non-coding SC neurons poorly predicted (*R*^2^ ~ 0.2) whisker position but provided moderate predictive power (*R*^2^ ~ 0.4) for locomotion speed (Fig 5F). Therefore, whisker angle is represented by a specialized population of neurons that are not simply driven by general changes in body movement or arousal. Importantly, many SC neurons simultaneously encoded whisking and locomotion. This multi-feature representation is potentially useful for controlling appetitive movements and may bestow animals with more seamless control over whisker-guided locomotor decisions [45,67–72].

The neural representation of whisking in SC neurons is potentially modulated by multiple brain areas [17,73]. The trigeminal nuclei and somatosensory cortex are the primary sources of whisker afference to the SC [18]. Activity in these brain regions is strongly correlated to the phase of whisker motion [23,25,74,75]. We found diverse forms (broad and narrow widths) of phase tuning in SC neurons, similar to previous observations in brainstem and cortex of awake animals. The motor cortex and cerebellum likely send whisker efference to the SC [13,20]. Neural activity in these brain regions is correlated to the amplitude and offset of whisker motion [29,31,33,76,77]. Unlike SC, motor cortex lacks a clear linear relationship between whisk offset and firing rate, indicating that offset tuning in SC could also be computed locally and/or inherited from the cerebellum. Importantly, projections from all four of these brain regions converge in a lateral zone of the SC that closely matches our recording site [19,16]. Therefore, the representation of absolute whisker position in SC neurons is likely built from the convergence of afferent and efferent inputs. Whether these converging inputs from high-order brain areas are necessary for self-motion tuning, or play more of a modulatory role, is unknown. A model of convergent signaling is supported by our whisker trimming results, which caused a modest disruption to slow self-motion tuning and a more frequent change to phase tuning (Fig 6). This is consistent with the sensory origin of phase tuning in the barrel cortex [74]. However, only 14% of neurons completely lost their phase tuning after trimming, suggesting the presence of efferent phase information, as in motor cortex [29]. However, residual forces on the follicle could have also contributed, as shown in the trigeminal ganglion [22]. Under anesthesia and during whisking induced by facial nerve stimulation, neurons in the trigeminal nuclei spike during the protraction phase of the whisk cycle [24,25,27]. In rats volitionally whisking, the phase preference of trigeminal neurons is broadly distributed between the protraction and retraction periods [26]. In our study, SC neurons also displayed a broad distribution of phase preferences, although there was a general preference for the retraction period. This preference for the retraction period could stem from efferent signals in the SC that are not present under anesthesia, which could be important for guiding interactions with the environment that depend on behavioral context [42]. The activity of most SC neurons was biased toward present or future kinematic features (Fig 3), in line with reafferent and efferent signaling, respectively. In conclusion, these data suggest that SC neurons combine multiple signals to build a comprehensive representation of whisker position that reflects past, present, and future states of self-motion. Such computations are critical for active sensing and computing the difference between the expected and real-world outcome of body movement [52,55,78,79].

The SC is known to play an important role in driving complex orienting movements. Microstimulation of its deep layer neurons shifts the gaze of primates by activating neck and eye muscles [80]. Similarly, stimulation of the SC in rodents drives head and whisker movements [17,40,81]. However, the SC does not operate in isolation. SC neurons provide excitatory input to the thalamus, ultimately augmenting whisker responses in S1 Fig and potentially changing the excitability of other cortical areas [17,82]. Interestingly, optogenetic activation of thalamus also induces head movements, and its neurons are co-tuned to head and whisker movement [83]. This opens the possibility that a loop between SC, thalamus, and cortex is important for executing whisker-guided head movements, perhaps analogous to visually guided body movements in primates [84]. The incorporation of thalamus and cortex into the sensorimotor loop could be important for controlling movements based on learned or innate associations [85–87]. Taken together, studies in head-free rodents in combination with our own work highlight the importance of freely moving experimental systems for revealing the neural computations supporting complex sensorimotor transformations [42,88–90]. Therefore, the physical constraints imposed on experimental subjects are an important consideration when assessing the information bandwidth of a brain area, as they could obscure diverse forms of mixed selectivity [91,92].

In our study, we discovered individual SC neurons that encoded both whisking and locomotion, hypothetically extending the role of the SC to the coordination of these coupled systems [44,45,68]. Anatomical and functional evidence supports this hypothesis, whereby SC neurons target brain regions involved in appetitive and exploratory locomotion as well as a spinal region involved in turning [66,70,93,94]. SC neurons also send excitatory projections to the nucleus that drives the whisking muscles [37,38,41]. In line with this anatomical connection, we discovered a subset of SC neurons that reliably spiked milliseconds before the start of whisker protraction, with greater spike rates preceding larger protractions (Fig 4I). Future research optogenetically identifying and manipulating SC neurons that target the brainstem, thalamus, or spinal cord would provide valuable insight into the collicular mechanisms of movement control [54,83,94–97].

## Methods

### Experimental model and subject details

Mice of CD-1 background of both sexes between the ages of 9 and 15 weeks were used for all experiments. The Purdue Institutional Animal Care and Use Committee (IACUC, 1801001676A004) and the Laboratory Animal Program approved all procedures. Mice were housed at room temperatures ranging between 68 and 79°F with humidity ranging between 40% and 60%. Mice were socially housed with five or less per cage and maintained in a reverse light–dark cycle (12:12 h). All experiments were conducted during the animal’s subjective night.

### Preparation for in vivo electrophysiology

Each mouse was fitted with a custom designed aluminum headplate for head fixation. Animals were anesthetized with isoflurane (3%–5%), while their body temperature and respiratory rate were monitored. To prevent eye dryness, artificial tears ointment was applied. The skin and fur on the skull were disinfected using 70% ethanol and betadine, and then incised with sterilized surgical instruments. Liquivet tissue adhesive and Metabond dental cement were applied to the skull and wound margins to secure the headplate. Buprenorphine was administered for pain relief as a post-operative analgesic.

One day before the recording session, mice were briefly (15–20 min) anesthetized to perform a craniotomy over the SC. A 1-mm diameter craniotomy was made (coordinates 4-mm posterior and 1.5-mm lateral from bregma) with a Robbins Instruments biopsy punch and sealed with Dowsil silicone gel and Body Double. The next day, mice were head-fixed on the free-spinning circular treadmill in the electrophysiology set-up. A 3-shank, 128-channel Neuronexus probe was inserted at the site of craniotomy using a NewScale micromanipulator. The probe was lowered through the cortex at 75 µm/min, searching for light-driven activity. After passing through the cortex, denoted by a total loss of spiking, the return of visual responses indicated electrode penetrance into the superficial layer of the SC (~1,000 µm below the cortical surface). The probe was further descended into the intermediate and deep layers, where whisker deflections elicited spiking. The receptive field of recorded neurons was mapped by deflecting individual whiskers and to identify which whiskers elicited the greatest response. Whiskers that did not elicit detectable changes in neural activity were trimmed off to improve whisker tracking. Recordings were targeted at the C-row whiskers. If the electrode missed the target, it was removed and re-positioned based on somatotopic coordinates. In most experiments, mice retained 3–5 whiskers across one or two rows; in a few experiments, only 1 or 2 whiskers were left intact.

### Mouse behavior

Two days after head plate implantation, mice were given the opportunity of run on a circular treadmill for 1 h daily, until they fully habituated to the apparatus and were able to maintain a steady running pace. This facilitated volitional running and active whisking on the day of the electrophysiological recording. An opaque flag was placed over the eye and white noise was broadcasted to minimize visual and auditory cues, respectively. To measure the distance traveled by the mouse on the wheel, the circular treadmill was attached to a rotary encoder. A trial was initiated after the mouse ran 200 cm on the treadmill, and ended after the mouse ran an additional 200 cm. Whiskers were imaged during the trial window. In 20% of trials, chosen in a random order, a stepper motor moved the tactile surface into the active whisking field. At the end of the tactile stimulus trial, the surface returned to its location outside the whisking field.

### Spike sorting

Spikes sorting was performed using the MATLAB package Kilosort2 and manually curated using Phy2 gui (https://github.com/cortex-lab/phy) [98]. During manual curation, spike clusters were assessed based on a set of standard quality metrics to ensure accurate single-unit isolation. Clusters were considered single units if they exhibited consistent and distinct spike waveforms and had a stable spatial location on the electrode array. Clusters with refractory period violations (spiking within 1 ms) as observed in cross-correlograms were discarded. Firing rate stability over time was also evaluated to distinguish between stable units and noise or transient artifacts. Only clusters meeting these criteria were classified as single units and included in subsequent analyses.

### Whisker imaging and tracking

Whiskers were imaged using a high-speed (500 fps) infrared camera (photonfocus DR1) through a mirror angled at 45° under IR illumination. Imaged videos were synchronized with neural spike data via external triggers using a National Instruments card and recorded on an Intan 512 controller. DeepLabCut was used to track whisker movement and curvature [46]. Whisker curvature was calculated from the three distal labels (of four total) on each whisker using Menger curvature. About 150 frames from each recording session were labeled manually, spanning diverse whisker positions. The neural network was trained for at least 200-k iterations, and the final labels were manually verified for accuracy.

### Extracting self-motion features

Whisker position was calculated as the angle between the frame’s vertical axis and the line joining a point on the mouse’s nose to a label on the whisker. The chosen label was the same between the intact and trimming conditions. Whisker envelope was generated by interpolating peaks and troughs of the whisker position using Akamai spline interpolation (see Fig 1B). Whisker offset was calculated as the upper bound of the envelope, while amplitude was the half width of the envelope.

Whisker phase was obtained by bandpass filtering the whisker position trace between 5 and 50 Hz (bandpass(), MATLAB) followed by calculating the Hilbert transform (hilbert(), MATLAB) and obtaining the angle from the complex value (angle(), MATLAB). A phase angle of 0° and −*π* was the most retracted angle, while *π* was the most protracted angle.

### Tuning curves

To create tuning curves for slow features such as locomotion speed, whisker offset, and amplitude, we segmented neuronal spike rates into 50-ms time intervals. Each feature’s total range was divided into 20 equal bins. For each feature bin, we calculated the mean and standard error of the spike rate. To find sharp transitions in whisker offset, findchangepts() function in MATLAB was used.

To obtain a neuron’s phase tuning, we generated spike triggered averages of whisker phase. The spike counts for each neuron were binned into 30° non-overlapping phase bins spanning from −*π* to +*π*, determined by the whisker phase angle at the time of each spike. To obtain neuronal firing rates as a function of whisker phase, spike counts in each phase bin were divided by the time spent at each angle. To test for significant phase tuning, we compared each neuron’s firing rate distribution to a uniform distribution using a Rayleigh test. The phase preference of each neuron was the circular mean of its phase tuning curve (circ_mean(), CircStat toolbox, MATLAB). The phase modulation depth of each neuron was calculated using the maximum, minimum, and mean firing rates of the phase tuning curve [23].


$$\text{modulation depth}=\left(\text{max}\left(FR\right)-\text{min}\left(FR\right)\right)/\text{mean}\left(FR\right).$$ (1)

### Linear-nonlinear Poisson (LNP) model analysis

To quantify the dependence of spiking on a feature, or a combination of features (locomotion speed, whisk offset, and whisk amplitude), we employed an LNP spiking GLM model, previously established in the field [48]. This approach is agnostic to tuning curve shape and robust to the interdependence of encoded variables. Considering the significant correlation between self-motion features observed in our study, this LNP approach is highly advantageous over traditional tuning curves (S2A Fig).

The LNP model estimates the spike rate (*N*^*t*^) of individual neurons at each time bin ‘*t*’ by taking an exponential sum of the weighted feature values. In the below equation, ‘*f*’ represents the features (Locomotion speed, Offset, Amplitude, Frequency), *X*_*f*_^*t*^ is a feature vector at time ‘*t*’, *W*_*f*_ is the learned weight that converts feature value to firing rate, and *dt* is the duration of time bin (50 ms). We binned neuron spike rates and self-motion features into 50-ms bins.


$$N^{t}*dt=e^{\underset{f}{\sum }(X_{f}^{t}*W_{f})},$$ (2)

where *X*_*f*_ is a binned feature occupancy vector. Each feature is binned into 20 bins that span the feature’s entire range. At each time point *t*, all bins are set to 0 except for the one that corresponds to the feature value at that time, which is set to 1. To determine the weight vector *W*_*f*_ for each neuron, we maximized the Poisson log-likelihood (LLH) of the observed spike train. The LLH of the observed spike train given the predicted firing rates was computed using the negative LLH for a Poisson distribution. Specifically, for each neuron, we calculated


$$\text{Negative LLH model}=\;\frac{1}{\text{Σ}n_{t}}{\text{Σ}}_{t}\left(r_{t}-n_{t}\log\left(r_{t}\right)+\log\left(n_{t}!\right)\right),$$

where *n*_*t*_ is the observed spike count at time bin *t*, *r*_*t*_ = exp^*f*()^(*X*_*t*_⋅*W*_*f*_) is the predicted firing rate from the model at time bin *t*, with *X*_*t*_ representing the binned feature vector and *W*_*f*_ being the learned weights. Model performance for each neuron was measured as relative to the mean firing rate model as


$$\text{Negative LLH meanFRmodel}=\;\frac{1}{\text{Σ}n_{t}}{\text{Σ}}_{t}\left(\bar{m}-n_{t}\log\left(m\right)+\log\left(n_{t}!\right)\right),$$

where *m* is the mean firing rate of the neuron.


$$\text{LLH }=\;\frac{-\text{negative LLH model }+\text{ negative LLH meanFRmodel}}{\log2}.$$

The model performance for each neuron was assessed as the LLH on held out data. We performed 10-fold cross-validation.

To identify which subset of features best explains spiking in each neuron, we performed a forward search approach (S3C and S3F Fig). This approach tested models with varying numbers of features: four 1-feature models (L, O, A, F), six 2-feature models (LO, LA, LF, OA, OF, AF), four 3-feature models (LAO, LAF, OAF, LOF), and one full model (LOAF). The process began by selecting the single-feature model with the highest performance. This model’s performance was then compared against all 2-feature models that included the chosen single feature. If the best-performing 2-feature model significantly outperformed the single-feature model, we then compared it to the 3-feature models, and so forth (S3E and S3F Fig). The chosen model was the one with the fewest features that did not show a significant improvement in prediction when additional features were added. If a neuron’s best model did not significantly outperform the mean firing rate model, it was labeled as unclassified. The same data used in the LNP model was used to calculate the mean firing rate of the neuron. We determined significance using a one-sided signed rank test with an *α* value of 0.05. For more details on the model, refer to https://github.com/GiocomoLab/ln-model-of-mec-neurons.

### Relative contribution of single features

We identified the contribution of a given feature by finding the difference in model performance (LLH increase) between the selected model and the model that contains all the features in the selected model except the given feature. For example, if a neuron’s selected model is LOA, then contribution of feature O is contrib(O) = LLH(LOA) − LLH(LA). Contributions were calculated for a given feature even when the given feature was not contained within the selected model for a neuron. For example, if a neuron’s selected model is LA, then the contribution of O is contrib(O) = LLH(LOA) – LLH(LA). Contributions of features that are not encoded in a neuron are usually very small, consistent with model selection procedure. In case, contribution of a given feature was negative (which could happen when the feature was not encoded by the neuron), then the negative values were reset to 0. After computing contributions of all three features, we normalized the contributions by making their sum equal 1.

### Time-shifted models

For each neuron, we fit 12 time-shifted LNP models. We binned the data into 25 ms, to accommodate for time shifts lower than 50 ms. The feature values were shifted by {−200, −150, −100, −75, −50, −25, 0, 25, 50, 75, 100, 150, 200} ms relative to the spike train. We plotted the LLH of each model against its time shift to create a temporal curve. Temporal bias was calculated as the difference between the area under the curve (AUC) for positive and negative time shifts, and then dividing this difference by the total area. The time shift with the highest LLH was identified as the preferred time shift.

### Temporal kernel analysis

To capture the influence of different time shifts on neuronal firing, we implemented a temporal GLM that simultaneously compares the contribution of multiple time shifts within a single model. This approach allowed us to derive temporal kernels for each feature, revealing distinct optimal time shifts for different behavioral features. Like our previous method, we modeled neurons with features derived from locomotion and whisker motion (locomotion speed, whisker offset, and whisker amplitude).

The model predicts the firing rate of a neuron at each 20-ms time bin *t* using a window of features spanning from *t* − 10 to *t* + 10 time bins, corresponding to a temporal window of −200 ms to +200 ms. This windowed approach captures both past and future whisker and locomotion influence on neuronal activity. The features were standardized by subtracting the mean and scaling to unit variance. A GLM with Ridge (L2) regularization was used for model fitting to avoid overfitting by penalizing large coefficients. Hyperparameter tuning was performed through 5-fold cross-validation on the training set to identify the optimal regularization strength. To validate the significance of the model, we conducted a permutation test by shifting the spike data randomly 1,000 times and refitting the model for each shuffled dataset. This procedure generated a null distribution of *R*^2^ values, representing the relationship between the features and spike rates under the assumption of no true association. We compared the original *R*^2^ value against this null distribution to assess significant relationship between neural spike rates and behavioral features. For each neuron, the feature with the highest sum of absolute coefficients (AUC) was identified as the preferred feature. The preferred time was determined as the specific time point (or lag) where the preferred feature has the maximum coefficient value within the lagged window.

### Self-motion decoder model

To determine if self-motion encoding in SC neurons support downstream decodability, we implemented a ridge regression model (Fig 5A, sklearn.linear_model.Ridge, Python). We decoded whisker position with a temporal resolution of 15 ms, matching the resolution of our binned spike rates. For decoding, we utilized a 165-ms time window, comprising 11 bins (5 preceding, 1 concurrent, and 5 following bins) of spike rate data. To fit a model, we conducted 10-fold cross-validation, and decoding performance was tested on 20% of held-out data. When decoding with a reduced number of neurons (from population total), we randomly selected self-motion encoding neurons, and the randomization was repeated 10 times to accurately gauge decoding performance. For more details of the code, refer the python version at https://github.com/sumachinta/body-position_decoding_model/tree/main.

### Simulated neuronal firing rates for self-motion decoding

Neuronal firing rates were simulated based on self-motion tuning curves. Firing rates were simulated in 15-ms time bins. The firing rate in a time bin *t* was generated by picking a random number from a Poisson distribution specified by the rate parameter *N*^*t*^ calculated from Eq 2 (poissrnd(), MATLAB). If a neuron did not encode a specific feature ‘*f*’, the weight ‘*W*_*f*_’ was set to 0. The simulated neurons were tuned to both single and multiple features, reflecting the diversity observed in real SC neuronal population, and 59% of the simulated neurons were trained on 1D tuning curves, while 41% of neurons were trained on 2/3D tuning curves.

To simulate neuron firing rates tuned to whisker phase, we divided the phase range into 12 bins and created a vector for each time bin ‘*t*’. In this vector, all bins were set to zeros except for the one corresponding to the current phase at time ‘*t*’, which is set to 1. Multiplying this vector with each neuron’s phase tuning curve gave us the rate parameter. The firing rate for each time bin was then determined using a random number from a Poisson distribution based on this rate parameter. The feature values to simulate spiking were taken from a randomly chosen recording session.

### Sensory reafference

To examine how reafference affects the coding of self-motion in SC neurons, we trimmed off the whiskers for the final ~25 trials of the recording session, sparing a portion of the whisker for tracking movement. To compare the change in self-motion tuning to slow kinematic variables, we fit a separate LNP model during the whisker trim period. We compared the model performances before and after whisker trimming by looking for significant differences in mean LLH over 10 folds of training (*t* test, 10-fold cross-validation). Neurons were categorized as modified if they exhibited a significant change in LLH, or unchanged if they did not. Neurons that had a selected LNP model before whisker trimming and no selected model after trim were categorized as lost units, while gain units had the opposite trend.

To measure the change in phase tuning, we checked if there was a significant correlation between the tuning curves before and after whisker trimming (using Pearson correlation coefficient corr(), MATLAB, with *α* =  0.05). Neurons that had a significant correlation between pre- and post-trim conditions were labeled as ‘no change’ units. Those without a significant correlation were identified as ‘modified’ units. Neurons that were significantly phase-tuned before whisker trimming (see ‘phase tuning curve’ section of Methods) but not significant post-trimming were categorized as ‘lost’ units. Conversely, neurons that were not phase-tuned pre-trimming but became significantly tuned post-trimming were classified as ‘gain’ units. Neuron’s spike waveforms were compared pre- and post-trimming to ensure that the units were stable across pre- and post-trimming conditions. Mean firing rates of neurons during pre- and post-trim condition were controlled for whisker position and locomotion speed. To match the whisker position and locomotion speed coverage, we generated 2D occupancy matrices where each bin is a combination of a position and speed bin. Finally, we matched position and speed coverage across two conditions by down-sampling data points from either condition so that occupancy time was matched for each bin.

## Supporting information

S1 Fig Electrode penetration and self-motion feature timing. (A) Dye labeling of the electrode shank at the recording site in the intermediate and deep layers of lateral SC overlaid with the outline of the coronal section taken from mouse brain atlas based on the stereotaxic coordinates of the recording site. (B) Distribution of locomotion speeds in recorded mice (12 mice). (C) Autocorrelation of whisker position reveals the fast and slow components. (D–F) Autocorrelation of whisker midpoint, amplitude, and phase. Midpoint and amplitude vary more slowly than phase. (G) Greatest depth of electrode penetration relative to the surface of the SC in each mouse. Each electrode shank spanned 840 μm of vertical space.
(EPS)

S2 Fig An LNP-based GLM to identify individual neuron selectivity to self-motion features. (A) Mean curves depicting the co-variation of locomotion speed and whisking dynamics, with Pearson correlation coefficients indicating the strength of association (1 mouse). (B) Schematic of a LNP framework model workflow, where kinematic features are weighted, summed, and passed through an exponential non-linearity to produce a Poisson-distributed spike estimate. (C) Example midpoint selective neuron and its spike prediction performance across single and multi-feature models. The forward search approach identifies the minimum feature model whose performance is significantly better than any simpler model. The selected model for this neuron is marked in red. (D) Overlay of the neuron’s actual firing rate (in gray) against the firing rate predicted by the selected model (in red), demonstrating the model’s fidelity in capturing the neuron’s response pattern. (E) Scatter plot comparing the performance of the ‘O’ model against the ‘L + O’ model. A p-value of 0.053 suggests no significant benefit from including ‘L’, favoring the simpler ‘O’ model (right-tailed signed-rank test). (F–H) same as C, D, and E for a 2-feature selective neuron.
(EPS)

S3 Fig Temporal preference of neurons according to kinematic feature preference. (A–C) Distribution of temporal biases for neurons whose best feature is locomotion speed, or offset, or amplitude, respectively (125 neurons for locomotion speed, 139 neurons for offset, and 83 neurons for amplitude respectively). (D–F) Distribution of preferred time shifts for neurons in A, B, and C, respectively. (G–I) Scatter plot of preferred time shift and temporal bias for neurons in A, B, and C, respectively.
(EPS)

S4 Fig Kinematic feature weights derived from a temporal kernel of past, present and future time lags. (A) Model weights for each of the slow features for three example neurons (organized by columns). (B) Absolute area under the curve of each feature weight distribution. (C) Tuning curves for the same example neurons. (D) Temporal preference of the neural population, organized according to the feature that had the largest weight calculated by total area under the curve. (E, F) Feature preference of the neural population, determined by either taking the entire AUC (E), or using only the zero time point (F). The individual values for panels D, E, and F are included in supplementary S1 _Data, Supplementary Figure 4 sheet.
(EPS)

S5 Fig Decoding self-motion. (A, B) Distribution of whisker position and locomotion speed decoding accuracies for 12 individual mice. One mouse with the very low decoding accuracy for locomotion speed has only 1 locomotion speed tuned neuron. (C) Whisker position decoding accuracy for 1 example mouse with increasing number of time bins (15 ms duration) in the self-motion decoder. (D) Locomotion speed decoding accuracy for 1 example mouse with increasing number of time bins (100 ms) in the self-motion decoder. (E) Whisker position decoding with simulated neurons with shuffled spikes. The individual values for panel A are included in supplementary S1 Data, Supplementary figure 5 sheet.
(EPS)

S1 Video Tracking whisking and locomotion while recording from a population of SC neurons. Video showing whisker position, locomotion speed, and neural spiking over time. A vertical line in each plot signifies the corresponding frame in the tracked video.
(MP4)

S2 Video Whisker tracking pre-trim. Video showing the whisker label that was used to track the position of the whisker over time in the pre-trimming condition.
(MP4)

S3 Video Whisker tracking post-trim. Video showing the same whisker label that was used to track the position of the whisker over time in the post-trimming condition.
(MP4)

S1 Data An excel file containing the individual data values that create the summary data figures in the manuscript. The excel file is referenced in the appropriate figure legends.
(XLSX)

## Abbreviations

AUC: area under the curve
IACUC: Institutional Animal Care and Use Committee
LLH: log-likelihood
LNP: linear-nonlinear Poisson
PCC: Pearson correlation coefficient
SC: superior colliculus
